# Phylogenetic Complexity of Morphologically Identified *Anopheles squamosus* in Southern Zambia

**DOI:** 10.3390/insects12020146

**Published:** 2021-02-08

**Authors:** Jordan E. Hoffman, Ilinca I. Ciubotariu, Limonty Simubali, Twig Mudenda, William J. Moss, Giovanna Carpi, Douglas E. Norris, Jennifer C. Stevenson, on behalf of Southern and Central Africa International Centers of Excellence for Malaria Research

**Affiliations:** 1 Department of Microbiology, Immunology and Pathology, Colorado State University, Fort Collins, CO 80523, USA; jordan.hoffman@colostate.edu; 2 Department of Biological Sciences, Purdue University, West Lafayette, IN 47907, USA; iciubota@purdue.edu (I.I.C.); gcarpi@purdue.edu (G.C.); 3 Macha Research Trust, Choma, Zambia; limonty.simubali@macharesearch.org (L.S.); twig.mudenda@macharesearch.org (T.M.); jennycstevenson80@gmail.com (J.C.S.); 4 Department of Epidemiology, Johns Hopkins Bloomberg School of Public Health, Baltimore, MD 21205, USA; wmoss1@jhu.edu; 5 The W. Harry Feinstone Department of Molecular Microbiology and Immunology, The Johns Hopkins Malaria Research Institute, Johns Hopkins Bloomberg School of Public Health, Baltimore, MD 21205, USA

**Keywords:** malaria, *Anopheles*, anopheline, residual transmission, understudied vector, mosquito

## Abstract

**Simple Summary:**

Despite dramatic reductions in malaria cases in the catchment area of Macha Hospital, Choma District, Southern Province in Zambia, prevalence has remained near 1–2% by RDT for the past several years. To investigate residual malaria transmission in the area, this study focuses on the relative abundance, foraging behavior, and phylogenetic relationships of *Anopheles squamosus* specimens. Morphological identification, molecular verification of anopheline species, and blood meal source were determined on individual samples. Data from these collections supported earlier studies demonstrating *An. squamosus* to be primarily exophagic and zoophilic, allowing them to evade current control measures. The phylogenetic relationships generated from the specimens in this study supported the hypothesis of cryptic taxa among *An. squamosus* specimens, which further emphasizes the importance of molecular identification of vectors. The primarily exophagic behavior of *An. squamosus* in these collections also highlights that indoor vector control strategies will not be sufficient for elimination of malaria in southern Zambia.

**Abstract:**

Despite dramatic reductions in malaria cases in the catchment area of Macha Hospital, Choma District, Southern Province in Zambia, prevalence has remained near 1–2% by RDT for the past several years. To investigate residual malaria transmission in the area, this study focuses on the relative abundance, foraging behavior, and phylogenetic relationships of *Anopheles squamosus* specimens. In 2011, higher than expected rates of anthropophily were observed among “zoophilic” *An. squamosus*, a species that had sporadically been found to contain *Plasmodium falciparum* sporozoites. The importance of *An. squamosus* in the region was reaffirmed in 2016 when *P. falciparum* sporozoites were detected in numerous *An. squamosus* specimens. This study analyzed Centers for Disease Control (CDC) light trap collections of adult mosquitoes from two collection schemes: one performed as part of a reactive-test-and-treat program and the second performed along a geographical transect. Morphological identification, molecular verification of anopheline species, and blood meal source were determined on individual samples. Data from these collections supported earlier studies demonstrating *An. squamosus* to be primarily exophagic and zoophilic, allowing them to evade current control measures. The phylogenetic relationships generated from the specimens in this study illustrate the existence of well supported clade structure among *An. squamosus* specimens, which further emphasizes the importance of molecular identification of vectors. The primarily exophagic behavior of *An. squamosus* in these collections also highlights that indoor vector control strategies will not be sufficient for elimination of malaria in southern Zambia.

## 1. Introduction

Persistence and re-emergence of malaria transmission are significant challenges to malaria control efforts around the world [1,2]. In some areas across Africa, vector species have shown a shift in behavior, with higher proportions foraging outdoors or at times when humans are outside bed nets [3,4,5]. At a population level, species composition is also changing in many areas, with species that were once considered abundant primary vectors now on the decline. In some cases, as well-recognized vectors are successfully controlled, other anopheline species previously perceived to have little or no role in malaria transmission dominate collections. The role of these species in malaria transmission is regionally variable, with species testing positive for *Plasmodium* sporozoites and displaying high rates of anthropophily in some areas and testing negative and avoiding humans in others [6,7,8]. Many of these understudied vectors evade existing indoor-targeted control methods by foraging and resting outdoors, and they often have appreciable rates of zoophily. Thus, they may contribute to the maintenance of malaria transmission even at low levels, making elimination goals unachievable with the current set of vector control tools. Describing the bionomics of these species is complicated by the fact that many of these mosquitoes may exist in undescribed species complexes [9]. Understanding the ecology and bionomics of these mosquitoes and determining their role in malaria transmission is increasingly important to reaching malaria reduction and elimination goals. In addition, elucidating the genetic structure of these understudied populations is critical to identifying species and associating behaviors that can be targeted for control. 

Malaria prevalence in Macha, Choma District, Southern Province, Zambia, has declined dramatically since 2004 [10]. As part of a national effort to control malaria, insecticide-treated nets (ITNs) were distributed in the area, artemisinin combination therapy (ACT) use was started in the early 2000s, and focused indoor residual spraying (IRS) was utilized. Despite these efforts, malaria prevalence as determined by rapid diagnostic test (RDTs) has remained stagnant around 1–2% in this area for the past several years [11]. Shifts in anopheline species composition have been demonstrated [12]. Prior to a drought in 2004–2005, the reported primary vectors were *An. arabiensis* and *An. funestus*. After the drought, populations of *An. arabiensis* persisted, while *An. funestus* have been absent [13]. Historically, the majority of collections conducted in Macha focused on indoor collections, and endophagic *An. arabiensis* mosquitoes were found to have a human blood index (HBI) as high as 0.923. Exophagic populations, however, have largely not been explored, and their zoophilic tendencies may reduce their vectorial capacity [8,13]. The shift to more exophagic and zoophilic populations, combined with the loss of *An. funestus*, has been hypothesized to have contributed to a decline in malaria prevalence in the region. The persistence of malaria in Macha, however, could also be attributed to the activity of understudied vectors [11]. Several species present in the Macha area have been implicated as alternate vectors in other regions of Africa, including *An. squamosus* [14,15,16]. 

*An. squamosus* was first described in 1901. As early as 1903, Theobald noted morphological differences between mosquitoes caught in South Africa and those caught in northern Zimbabwe that he still recorded as *An. squamosus* [17,18,19], suggesting the existence of a possible species group or complex. *An. squamosus* has been found to have a broad distribution across sub-Saharan Africa and has even been found on the Arabian Peninsula [20,21]. Adults of the species have been shown to be primarily exophilic and exophagic. An apparent lack of impact of IRS on *An. squamosus* vector counts indicates that it is a strongly exophilic species across the continent [22,23,24,25,26,27,28]. Foraging behavior, however, seems to vary regionally. In an increasing number of reports, *An. squamosus* is caught more often indoors by CDC light traps and human landing catches (HLCs) than recognized malaria vectors [22,23,24,29,30,31,32,33]. Although much of the data supports the strong zoophilic behavior of *An. squamosus* [33,34,35,36], some collections have exhibited opportunistic foraging behaviors [33,37] and others high rates of anthropophily [6,8]. Gillies detected sporozoites in *An. squamosus* in Tanzania in the 1960s, illustrating the potential role for the species in malaria transmission [15,38]. 

In 2010, *An. squamosus* from the Macha area were reported to be unexpectedly anthropophilic, and in 2015, several specimens tested positive for *P. falciparum* sporozoites [6,7]. *Plasmodium* sporozoites have been detected in the salivary glands of *An. squamosus* in four countries, with anthropophilic behavior demonstrated in both Zambia and in Madagascar, but the species has not been reported to play an important role in malaria transmission to date [6,8,15,39,40,41]. Research on the bionomics and genetic diversity of *An. squamosus* therefore, has been minimal. This study focused on *An. squamosus* samples collected as part of two research projects spanning different spatiotemporal collections in Southern Province, Zambia. The goal was to assess the relative abundance of *An. squamosus* to other anophelines, anthropophily rates, and *An. squamosus* genetic diversity. These findings contribute to our knowledge and understanding of residual malaria transmission in southern Zambia.

## 2. Materials and Methods

### 2.1. Study Area and Mosquito Collections

**Figure 1:**
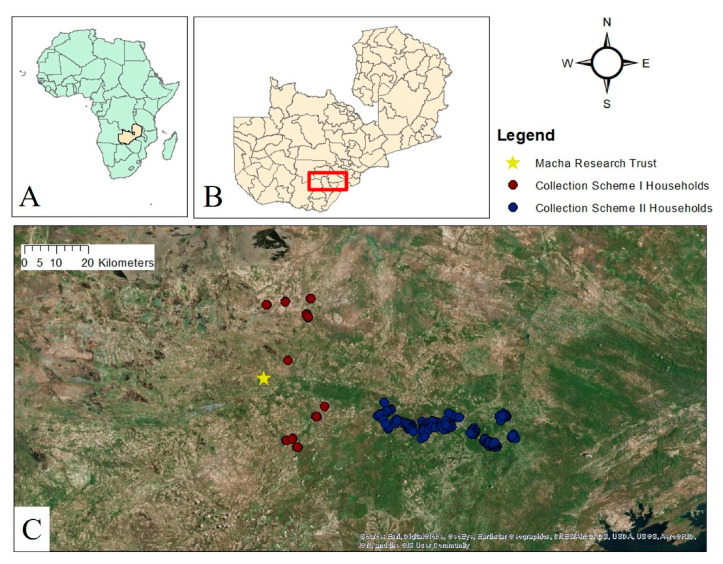
(**A**) Map of Africa; Zambia highlighted in light yellow. (**B**) Map of Zambia and its districts; study area is enclosed in red. (**C**) Satellite imagery of study area delineating households included in this study. Households are colored according to the collection scheme under which they were sampled.

All study households (*n* = 150) were located within an 81 km radius of Macha Research Trust (MRT), located at an elevation of 1100 m above sea level at 16.39292° S, 26.79061° E, within Choma District in Zambia’s Southern Province (Figure 1). The ecotype around the field station is primarily miombo woodland. The region experiences three seasons: a cool dry season (typically from May through July), a hot dry season (typically from August through October), and a rainy season (typically from November through April).

Mosquitoes and associated household data were sourced from two studies conducted by the Southern and Central Africa International Centers of Excellence for Malaria Research (ICEMR) with different sampling strategies. The first collection scheme involved collecting mosquitoes in homesteads (*n* = 23) identified as part of a reactive-test-and-treat program, where traps were set at index case homes and nearby secondary homes [42]. All collections in this scheme were conducted between December 2017 and June 2018 for a total of 96 trap nights. The second set of samples was derived from randomly selected households (*n* = 127) sampled between May and July 2018 along a transect running west-east from Macha towards Lake Kariba in Southern Province, Zambia (Stevenson, unpublished). All mosquito collections were performed between the hours of 6 p.m. and 6 a.m. using miniature CDC light traps set either indoors next to humans sleeping under a bed net or outdoors next to animal pens for a total of 251 trap nights. 

### 2.2. Sample Processing and Morphological Identification

After collection, traps were transported to MRT, and mosquitoes were killed by freezing. Anophelines were then separated by sex. Females were morphologically identified using a dichotomous key [43] and placed individually into 0.6 mL tubes with silica gel desiccant and cotton plug for storage at room temperature until further processing. All samples were then stored at −80 °C. A subset of the samples (*n* = 326) was transported to the laboratory at Johns Hopkins Bloomberg School of Public Health (JHSPH) in Maryland, USA, for more extensive molecular and genetic analysis, while the remaining samples (*n* = 3247) were analyzed in the laboratory at MRT. 

### 2.3. Mosquito Species Assignment

Mosquito abdomens were homogenized individually, and genomic DNA was extracted from each homogenate using a modified salt extraction [44]. To confirm species, all samples were first run on a PCR targeting the variable internal transcribed spacer 2 (ITS2) region that distinguishes between several species or groups of anophelines [40]. Samples whose product size from the ITS2 PCR was 600 bp were then run on a PCR targeting the ribosomal DNA intergenic spacer region designed to further distinguish members of the *An. gambiae* species complex as previously described [45]. Due to consistent failure of *An. squamosus* samples to amplify with the original ITS2 primers [35], two additional primers were included that specifically targeted a 330 bp fragment of the cytochrome oxidase I (COI) gene of *An. squamosus* (Jones, unpublished): SQFor405 (5′- CCA TTT CCA TTA TGT CCT ATC TAT AGG -3′) and SQRev707 (5′- GGG AAA GCA GGA GTT CGT TGA G- 3′). Each 25 µL reaction contained 2.5 µL of 10X PCR buffer, 200 µM of each dNTP, 30 pmol of each primer (ITS2A, ITS2B, SQFor405, SQRev707), 2.0 units of *Taq* polymerase, 1.0 µL of DNA template, and remaining volume with nuclease-free water. Products were amplified under the following thermocycler (MultiGene™ OptiMax Thermal Cycler, Labnet International, Inc., Edison, NJ, USA) conditions: 94 °C for 2 min, 40 cycles of denaturation at 94 °C for 30 s, annealing at 50 °C for 30 s, and extension at 72 °C for 40 s, with a final extension at 72 °C for 10 min. 

Of the sample set brought to JHSPH, a subset was run on a PCR that amplifies the Barcode of Life Database (BOLD) molecular target of the cytochrome *c* oxidase subunit I (COI) gene [40]. Any samples that were not assigned to species using the ITS2 PCR with or without the addition of the “*An. squamosus* primers” were included in this subset. Additional samples were included in this analysis to ensure samples sequenced were representative of the larger dataset by selecting every 10th sample from the dataset. The GPS coordinates for each sample were then plotted to ensure the selected samples were spatially representative. This fragment of the COI gene was amplified using the previously described LCO1490 and HCO2198 primers, and each 25 μL PCR reaction had the same mixture components previously used and identical thermocycler conditions [40,46]. All PCR products were visualized by electrophoresis on 2% agarose gels stained with ethidium bromide. All samples that produced the ~700 bp band were then purified using a QIAquick PCR Purification Kit (Qiagen, Hilden, Germany) and sent to the Johns Hopkins Medical Institutions (JHMI) Synthesis and Sequencing Facility for Sanger sequencing. Forward and reverse sequences obtained from the JHMI facility were imported into Geneious (Biomatters, Auckland, New Zealand) version 11.1.5 (https://www.geneious.com) and trimmed to remove low-Phred quality ends. Forward and reverse sequences were pairwise aligned to create one consensus sequence for each sample. Each individual sample sequence was compared against the NCBI database using BLASTn, and samples were identified as a specific species when there was a minimum nucleotide identity of 95% and a significant E-value <1 × 10^−5^. All COI consensus sequences were trimmed to a final length of 671 bp and submitted to GenBank. These sequences were further utilized for the phylogenetic analysis. 

### 2.4. Host Blood Meal Identification

To determine blood meal source, samples brought to JHSPH were run on a PCR targeting the 12S ribosomal RNA gene that signals presence of a blood meal. This PCR used two primers, a universal forward and a universal reverse, to detect vertebrate DNA: UNIFic (5′- GGA TTA GAT ACC CCA CTA TGC -3′) and UNIRic (5′- GCT GAA GAT GGC GGT ATA TAG -3′). Each 25 µL reaction contained 2.5 µL of 10X PCR buffer, 200 µL of each dNTP, 0.3 µL of the primers, 2.0 units of *Taq* polymerase, 1.0 µL of DNA template, and nuclease-free water comprising the remaining volume. Genomic DNA was amplified under the following thermocycler conditions: 5 min at 94 °C, 35 cycles of denaturation at 94 °C for 30 s, annealing at 51 °C for 30 s, and extension at 72 °C for 30 s, and 7 min for final extension at 72 °C. The same BLASTn method and criteria described above for mosquito species assignment from sequences was used for the assignment and determination of blood meal host.

### 2.5. Phylogenetic Analysis

A spatiotemporally representative selection of the COI consensus sequences generated (*n* = 28) were aligned with representative sequences (*n* = 27) of the same COI target fragment from NCBI, using the MUSCLE algorithm to generate a multiple alignment. The resulting alignment was then used to generate a Maximum Likelihood (ML) phylogenetic tree using Mega X [47,48]. The tree was constructed using a Generalized Time Reversible (GTR) model of nucleotide substitution, and 1000 bootstrap replications were used for branch support. One final tree showing the highest log likelihood for the COI target was selected for inclusion in this study.

## 3. Results

### 3.1. Species Composition

#### 3.1.1. Collection Scheme I: Reactive-Test-and-Treat Program

From December 2017 until June 2018, a total of 941 anophelines were collected under the reactive-test-and-treat project, 751 outdoors and 190 indoors, with at least seven anopheline species molecularly verified. *Anopheles squamosus* dominated species composition outdoors, representing 41.3% of outdoor collections (*n* = 311) (Table 1). Other species collected outdoors included *An. rufipes* (24.2%, *n* = 182), *An. quadriannulatus* (9.2%, *n* = 69), *An. arabiensis* (5.3%, *n* = 40), *An. coustani* (5.0%, *n* = 38), *An. longipalpis* (3.6%, *n* = 27) and *An. pretoriensis* (0.1%, *n* = 1). The remaining 11.3% (*n* = 83) of specimens collected outdoors remained molecularly unverifiable. Of those, 31.8% were morphologically identified as *An. rufipes* (*n* = 27), 27.1% as *An. gambiae* s.l. (*n* = 23), 23.5% as *An. squamosus* (*n* = 20), and 1.2% as *An. pretoriensis* (*n* = 1). Fourteen samples (1.9% of all outdoor specimens) were not identifiable by morphology or molecular techniques. 

Although 63% (*n* = 61) of traps were set indoors, only 25.2% of anophelines in collection scheme I were collected indoors (Table 1). *Anopheles arabiensis* dominated indoor mosquitoes, representing 43.2% (*n* = 82) of indoor collections. *An. squamosus*, however, was the second most abundant, making up 28.4% (*n* = 54) of indoor collections. Other species collected indoors included *An. longipalpis* (3.7%, *n* = 7), *An. coustani* (3.2%, *n* = 6), *An. quadriannulatus* (2.1%, *n* = 4), and *An. rufipes* (1.6%, *n* = 3). The remaining 17.9% (*n* = 34) of specimens collected indoors were molecularly unverifiable. Of those, all were morphologically identified as *An. gambiae* s.l. (*n* = 34). 

#### 3.1.2. Collection Scheme II: Transect

In May and June of 2018, 2632 anophelines were collected, 2463 outdoors and 169 indoors, and morphologically identified for the transect survey. Outdoors, *An. squamosus* represented 7.9% of collections (*n* = 194) (Table 2). Other species included *An. rufipes* (29.7%, *n* = 731), *An. coustani* (9.5%, *n* = 235), *An. gambiae* s.l. (7.1%, *n* = 174), *An. funestus* s.l. (6.2%, *n* = 152), *An. pretoriensis* (3.7%, *n* = 92), and *An. longipalpis* (2.4%, *n* = 60). *An. brunnipes*, *An. dancalicus*, *An. hancocki/brohieri*, *An. machardyi*, *An. maculipalpis*, and *An. theileri* together made up less than 1% of collections (*n* = 24). A remaining 32.4% (*n* = 799) were unable to be morphologically identified. 

Although 51% (*n* = 127) of traps were set indoors, only 6.4% (*n* = 169) of anophelines in collection scheme II were collected indoors (Table 2). *An. gambiae* s.l. dominated indoors, representing 39.0% (*n* = 66) of collections. Other species collected indoors included *An. rufipes* (13.6%, *n* = 23), *An. funestus* s.l. (4.1%, *n* = 7), *An. coustani* (3.6%, *n* = 6), *An. squamosus* (3.6%, *n* = 6), and *An. longipalpis* (1.8%, *n* = 3). A remaining 33.7% (*n* = 57) were unable to be morphologically identified. The high rate of failure of morphological identification in both indoor and outdoor collections was due largely to a high rate of specimen damage; 95% of unidentified samples were recorded as damaged during collection. 

### 3.2. Host Identification 

Of the subset of anophelines brought to JHSPH (*n* = 326), 323 were tested to identify the blood meal host. The remaining three specimens in the subset were males and thus were not included in this analysis. Blood meals were detected in 61 of the 109 visibly blooded anophelines and in 32 of the 214 anophelines that were not visibly blooded following amplification of the 12S region of the ribosomal RNA and subsequent Sanger sequencing (Table 3). Human blood meals were only identified in *An. arabiensis* (*n* = 3). The 3 *An. arabiensis* positive for human blood meals were collected indoors, whereas all other anophelines with identifiable blood meals were collected outdoors. Of the *An. squamosus* samples with detectable blood meals, 67.1% (*n* = 53) had fed on goat and 32.9% (*n* = 26) on cow. 

### 3.3. Species Assignment and Phylogenetic Analyses

Of the sample set brought to JHSPH for additional molecular analysis (*n* = 326), Sanger sequencing of the COI BOLD target was performed on a subset of samples. 28 samples (Table 4) were selected for phylogenetic analysis based on quality of the alignment and ensuring spatiotemporal representation of the subsample. Species identification for each sample relied on a threshold of 95% or greater query cover and nucleotide identity agreement. 

Of note, for seven of the samples from the transect collection, a fragment that appeared larger than 1000 bp was produced from the ITS2 PCR, a band size as yet undescribed in the protocol. All seven samples were morphologically identified as *An. squamosus*. Comparison of sequences of the Barcode of Life COI PCR target from these specimens with the NCBI database matched most closely with *An. species 15*.

**Figure 2:**
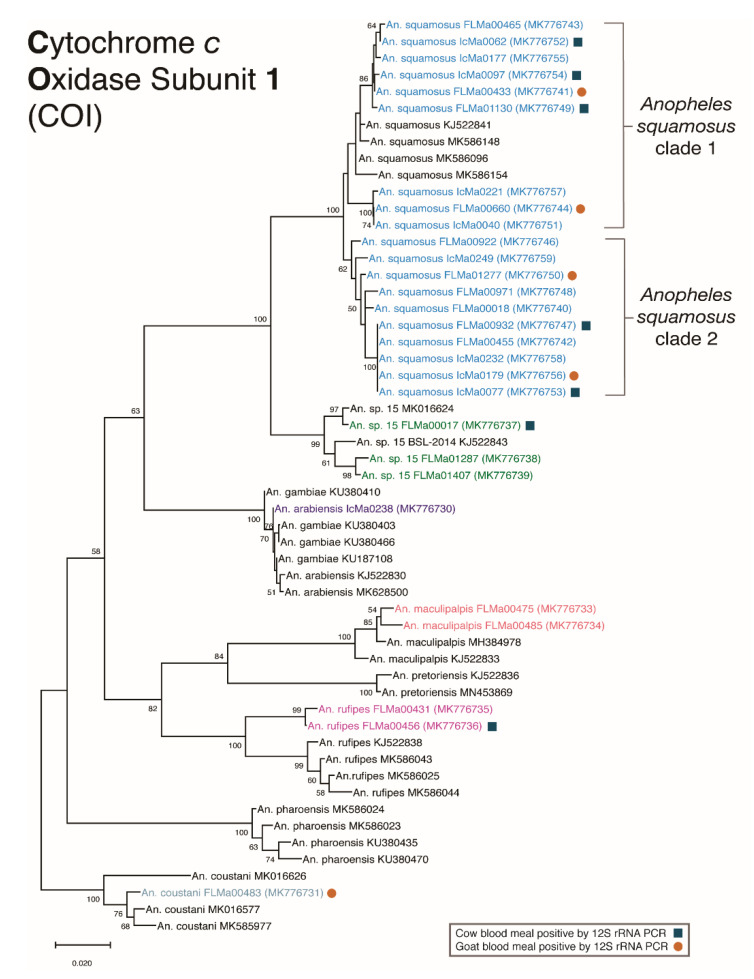
Cytochrome oxidase subunit I (COI) Maximum Likelihood tree. Bootstrap probabilities of branches are displayed at the nodes. Samples from this study are displayed in color by species, and samples from the NCBI database are shown in black. Evolutionary analyses were conducted in MEGA X. The phylogenetic tree was created using the Maximum Likelihood method and General Time Reversible substitution model, with 1000 bootstrap replicates. The above tree with the highest log likelihood (−3804.22) is drawn to scale. This analysis contained a total of 55 total samples, including 28 from this study and 27 representative sequences from NCBI BLASTn with labeled accession numbers.

The Maximum Likelihood (ML) phylogenetic tree was constructed using a 671 base pair fragment of the COI BOLD region for a representative subset of samples from both collection schemes (*n* = 28) and previously published sequences (*n* = 27) (Figure 2). The resulting phylogenetic tree showed that all samples morphologically and molecularly identified as *An. squamosus* grouped together. The ML tree revealed two distinct clades among *An. squamosus* specimens with 100% bootstrap support. Moreover, samples from this study that were molecularly identified to be *An. sp*. *15* grouped together with previously identified *An. sp*. *15* samples from the NCBI database. These samples formed a strongly supported group that as expected separated from *An. squamosus* (bootstrap of 100). The remaining sequences molecularly identified as other species (*An. arabiensis*, *An. maculipalpis*, *An. rufipes*, and *An. coustani*) clustered together with previously published sequences from the respective species from NCBI. *Anopheles rufipes* samples from this study did form a separate clade from *An. rufipes* reference samples, which might reflect geography, as the reference samples were collected in Kenya and Mali. Samples from both collection schemes were approximately evenly distributed between the two *An. squamosus* groups. Blood meal host and whether captured indoors or outdoors were also evenly distributed between the two clades. 

## 4. Discussion

This study evaluated relative *An. squamosus* abundance in field collections, foraging behavior, and phylogenetic relationships in an area of low, yet sustained residual malaria transmission in southern Zambia. *Anopheles squamosus* was previously found to demonstrate both unexpected anthropophily and carriage of *P. falciparum* sporozoites in the area, implicating it as an under-recognized vector [6,7]. In this study, *An. squamosus* was the most abundant species outdoors and second most abundant indoors among the reactive test-and-treat collections. In the transect collections, *An. squamosus* was less abundant overall but still numerous outdoors. The differences between the collection schemes could be spatiotemporal, as data from past collections suggested the abundance of *An. squamosus* varied with season of collection [31] and collection method. Collections using UV light traps and barrier screens outdoors reported *An. squamosus* comprising 40% of collections [7], whereas collections using human landing catches indoors and outdoors in addition to CDC light traps found *An. squamosus* comprised 26% of collections overall [6]. Despite these differences, the consistent abundance of *An. squamosus* throughout the rainy season and continued presence into the dry season highlight its potential role in maintaining malaria transmission in the absence of vector control strategies that would target this species.

Despite the preference for exophagy reflected in the literature, *An. squamosus* has been found to forage indoors at these collection sites. In this study, *An. squamosus* comprised 30% and 3.6% of indoor specimens from collection schemes I and II, respectively. This finding is consistent with previous studies in southern Zambia where *An. squamosus* comprised 10–20% of indoor collections depending on trapping method [6]. Prior studies have provided evidence of anthropophily for this species [6,8]. Human DNA was not detected in the blood meals of any *An. squamosus* analyzed in this study. This may be due to the limited number of mosquitoes in the subset containing identifiable blood meals (31%, *n* = 93), that most specimens in the subset were collected near livestock pens (90%, *n* = 292), or that the molecular assay based on Sanger sequencing used in this study lacked the ability to differentiate mixed host blood meals and their relative proportions. Significant rates of anthropophily have been reported for *An. squamosus* in Madagascar despite a high proportion of samples containing blood meals from multiple hosts [8,49]. However, the rates of anthropophily can also vary significantly over time, as *An. squamosus* in Madagascar was historically reported to be zoophilic before becoming primarily anthropophilic in the 1950s and has since been reported as zoophilic once more [8,34,36]. Shifts in foraging behavior warrant further investigation, but the composite data strongly suggest that *An. squamosus* is primarily zoophilic and opportunistically feeds on humans. 

The resulting topology of the BOLD COI phylogenetic analysis of all *An. squamosus* samples from this study (Figure 2) reinforces the previously described hypothesis that *An. squamosus* is a species complex [6,7]. *An. squamosus* is morphologically indistinguishable from *An. cydippis,* a member of the *An. squamosus* group, in the adult stage [17,43], the only stage of specimens available for this study. Although larvae of these two species can be easily distinguished, where *An. cydippis* appears in the literature, it is often referred to as a “variety” of *An. squamosus* [50]. Mass spectrometry has revealed detectable differences between the two, but there have been no studies thus far investigating the genetic differences between the two species [51]. Figure 2 illustrates two well supported groups among specimens morphologically and molecularly identified as *An. squamosus,* as has been demonstrated in previous literature from Zambia [6,7]. The phylogenetic analysis also reveals the relatively close, yet distinct, genetic relationship of *An. sp. 15* to specimens recognized as *An. squamosus*. All *An. sp. 15* specimens in this study, as well as in a published study from northern Zambia, were morphologically identified as *An. squamosus*. Whereas *An. squamosus* specimens fail to amplify using standard ITS2 primers, specimens identified as *An. sp. 15* produce a consistently large band outside the scope described for this molecular tool. While inadequate for taxonomic identification, these collective data help to differentiate *An. sp. 15* from *An. squamosus*. Although sequence of the BOLD COI fragment reliably differentiates between these taxa, there is not yet enough genetic data from these or related species to determine the complete phylogenetic relationships between these taxa. Similarly, as all our samples were collected as adults, and as there are no known molecular tools nor sequence data for differentiating *An. squamosus* and *An. cydippis*, it is impossible to know with certainty whether *An. cydippis* is represented in our data. Future studies should be designed to include collection, morphology, full genomes, and genomics of all life stages [52].

## 5. Conclusions

These data demonstrate that anophelines recognized as *An. squamosus* may be playing a role in malaria transmission in pre-elimination southern Zambia. Although the preference for zoophily is reinforced by this study, the dominance of *An. squamosus* in collections in and around human dwellings raises concerns. The strong zoophily, exophagy, and exophily of *An. squamosus* suggest that the traditional methods of vector control such as IRS and ITN use will likely not be effective against this vector. The WHO does not currently recommend outdoor control strategies due to the lack of convincing evidence of their efficacy [53]. If the global community fails to develop effective outdoor vector control strategies, and if Zambia and other regions in sub-Saharan Africa do not adapt their vector control strategies to include such outdoor control methods, malaria elimination may be difficult to achieve. 

## Figures and Tables

**Table 1:** Molecular species composition of the reactive test-and-treat collections from December 2017–June 2018.

Species	Number Collected	Mean Number per Trap Night	% of Total Collection
Outdoors (35 trap nights)			
*An. squamosus*	311	8.9	41.3
*An. rufipes*	182	5.2	24.2
*An. quadriannulatus*	69	2.0	9.2
*An. arabiensis*	40	1.1	5.3
*An. coustani*	38	1.1	5.0
*An. longipalpis*	27	0.8	3.6
*An. pretoriensis*	1	0.03	0.1
Unverified	83	2.4	11.1
Total	751	21.5	100.0
Indoors (61 trap nights)			
*An. arabiensis*	82	1.3	43.2
*An. squamosus*	54	0.9	28.4
*An. longipalpis*	7	0.1	3.7
*An. coustani*	6	0.1	3.2
*An. quadriannulatus*	4	0.07	2.1
*An. rufipes*	3	0.05	1.6
Unverified	34	0.6	17.9
Total	190	3.1	100.0

**Table 2:** Morphological species composition of the transect collections from May 2018–June 2018.

Morphological Species	Number Collected	Mean Number per Trap Night	% of Total Collection
Outdoors (124 trap nights)			
*An. rufipes*	731	5.9	29.7
*An. coustani*	235	1.9	9.5
*An. squamosus*	194	1.6	7.9
*An. gambiae* s.l.	174	1.4	7.1
*An. funestus* s.l.	152	1.2	6.2
*An. pretoriensis*	92	0.7	3.7
*An. longipalpis*	60	0.5	2.4
*An. maculipalpis*	15	0.1	0.6
*An. theileri*	5	0.04	0.2
*An. brunnipes*	1	0.01	0.04
*An. dancalicus*	1	0.01	0.04
*An. hancocki/brohieri*	1	0.01	0.04
*An. machardyi*	1	0.01	0.04
Unverified	799	6.4	32.4
Total	2463	19.9	100.0
Indoors (127 trap nights)			
*An. gambiae* s.l.	66	0.5	39.0
*An. rufipes*	23	0.2	13.6
*An. funestus* s.l.	7	0.1	4.1
*An. squamosus*	6	0.05	3.6
*An. coustani*	6	0.05	3.6
*An. longipalpis*	3	0.02	1.8
Male	1	0.01	0.6
Unverified	57	0.4	33.7
Total	169	1.3	100.0

**Table 3:** Identification of blood meals using 12S rRNA PCR.

	Human	Cow	Goat
*An. squamosus*		26	53
*An. sp. 15*		1	3
*An. arabiensis*	3		
*An. rufipes*		1	2
*An. coustani*			2
*An. maculipalpis*			1
*An. quadriannulatus*			1

**Table 4:** GenBank accession numbers, morphological and molecular species identifications, month/season and location of collection, and host choice for all specimens included in the phylogenetic analysis in this study. *Anopheles squamosus* clades are also listed for all molecularly confirmed *An. squamosus* specimens. All samples were collected in 2018.

Specimen ID	Molecular Species Identification	Morphological Species Identification	Accession Number	Month/Season of Collection	Collection Scheme/Trap Location	Host	*An.* *Squamosus* Clade
IcMa0238	*An. arabiensis*	*An. dancalicus*	MK776730	February/Rainy	I/Indoors		
FLMa00483	*An. coustani*	*An. squamosus*	MK776731	May/Cool Dry	II/Goat Pen	Goat	
FLMa00475	*An. maculipalpis*	*An. squamosus*	MK776733	May/Cool Dry	II/Goat Pen		
FLMa00485	*An. maculipalpis*	*An. squamosus*	MK776734	May/Cool Dry	II/Goat Pen		
FLMa00431	*An. rufipes*	*An. squamosus*	MK776735	May/Cool Dry	II/Goat Pen		
FLMa00456	*An. rufipes*	*An. squamosus*	MK776736	May/Cool Dry	II/Cattle Pen	Cow	
FLMa00017	*An. sp. 15*	*An. squamosus*	MK776737	May/Cool Dry	II/Cattle Pen	Cow	
FLMa01287	*An. sp. 15*	*An. squamosus*	MK776738	May/Cool Dry	II/Goat Pen		
FLMa01407	*An. sp. 15*	*An. squamosus*	MK776739	May/Cool Dry	II/Goat Pen		
FLMa00433	*An. squamosus*	*An. squamosus*	MK776741	May/Cool Dry	II/Goat Pen	Goat	1
FLMa00465	*An. squamosus*	*An. squamosus*	MK776743	May/Cool Dry	II/Indoors		1
FLMa00660	*An. squamosus*	*An. squamosus*	MK776744	May/Cool Dry	II/Goat Pen	Goat	1
FLMa01130	*An. squamosus*	*An. squamosus*	MK776749	May/Cool Dry	II/Goat Pen	Cow	1
IcMa0040	*An. squamosus*	*An. pretoriensis*	MK776751	February/Rainy	I/Cattle Pen		1
IcMa0062	*An. squamosus*	*An. squamosus*	MK776752	February/Rainy	I/Cattle Pen	Cow	1
IcMa0097	*An. squamosus*	*An. squamosus*	MK776754	February/Rainy	I/Cattle Pen	Cow	1
IcMa0177	*An. squamosus*	*An. squamosus*	MK776755	February/Rainy	I/Goat Pen		1
IcMa0221	*An. squamosus*	*An. squamosus*	MK776757	February/Rainy	I/Indoors		1
FLMa00018	*An. squamosus*	*An. squamosus*	MK776740	May/Cool Dry	II/Cattle Pen		2
FLMa00455	*An. squamosus*	*An. squamosus*	MK776742	May/Cool Dry	II/Cattle Pen		2
FLMa00922	*An. squamosus*	*An. squamosus*	MK776746	May/Cool Dry	II/Goat Pen		2
FLMa00932	*An. squamosus*	*An. squamosus*	MK776747	May/Cool Dry	II/Goat Pen	Cow	2
FLMa00971	*An. squamosus*	*An. squamosus*	MK776748	May/Cool Dry	II/Goat Pen		2
FLMa01277	*An. squamosus*	*An. squamosus*	MK776750	May/Cool Dry	II/Goat Pen	Goat	2
IcMa0077	*An. squamosus*	*An. squamosus*	MK776753	February/Rainy	I/Cattle Pen	Cow	2
IcMa0179	*An. squamosus*	*An. squamosus*	MK776756	February/Rainy	I/Goat Pen	Goat	2
IcMa0232	*An. squamosus*	*An. squamosus*	MK776758	February/Rainy	I/Indoors		2
IcMa0249	*An. squamosus*	Male	MK776759	February/Rainy	I/Indoors		2

## Data Availability

The genetic data presented in this study are publicly available on GenBank and accession numbers are reported in Table 4. Other data are available on request from the corresponding author due to privacy and ethical restrictions.
